# The deep(er) roots of Eukaryotes and Akaryotes

**DOI:** 10.12688/f1000research.22338.2

**Published:** 2020-06-22

**Authors:** Ajith Harish, David Morrison

**Affiliations:** 1 Independent Researcher, Uppsala, 756 57, Sweden; 2 Department of Organismal Biology, Systematic Biology, Uppsala University, Uppsala, 752 36, Sweden

**Keywords:** Asgard archaea, 2D, tree of life, LUCA, phylogenomics, nonstationary, rooting, eukaryogenesis

## Abstract

**Background: **Locating the root node of the “tree of life” (ToL) is one of the hardest problems in phylogenetics, given the time depth. The root-node, or the universal common ancestor (UCA), groups descendants into organismal clades/domains. Two notable variants of the two-domains ToL (2D-ToL) have gained support recently. One 2D-ToL posits that eukaryotes (organisms with nuclei) and akaryotes (organisms without nuclei) are sister clades that diverged from the UCA, and that Asgard archaea are sister to other archaea. The other 2D-ToL proposes that eukaryotes emerged from within archaea and places Asgard archaea as sister to eukaryotes. Williams *et al*. ( *Nature Ecol. Evol.* 4: 138–147; 2020) re-evaluated the data and methods that support the competing two-domains proposals and concluded that eukaryotes are the closest relatives of Asgard archaea.

**Critique: **The poor resolution of the archaea in their analysis, despite employing amino acid alignments from thousands of proteins and the best-fitting substitution models, contradicts their conclusions. We argue that they overlooked important aspects of estimating evolutionary relatedness and assessing phylogenetic signal in empirical data. Which 2D-ToL is better supported depends on which kind of molecular features are better for resolving common ancestors at the roots of clades – protein-domains or their component amino acids. We focus on phylogenetic character reconstructions necessary to describe the UCA or its closest descendants in the absence of reliable fossils.

** **

**Clarifications: **It is well known that different character types present different perspectives on evolutionary history that relate to different phylogenetic depths. We show that ****protein structural-domains support more reliable phylogenetic reconstructions of deep-diverging clades in the ToL. Accordingly, Eukaryotes and Akaryotes are better supported clades in a 2D-ToL.

## Background

**Figure 1.**
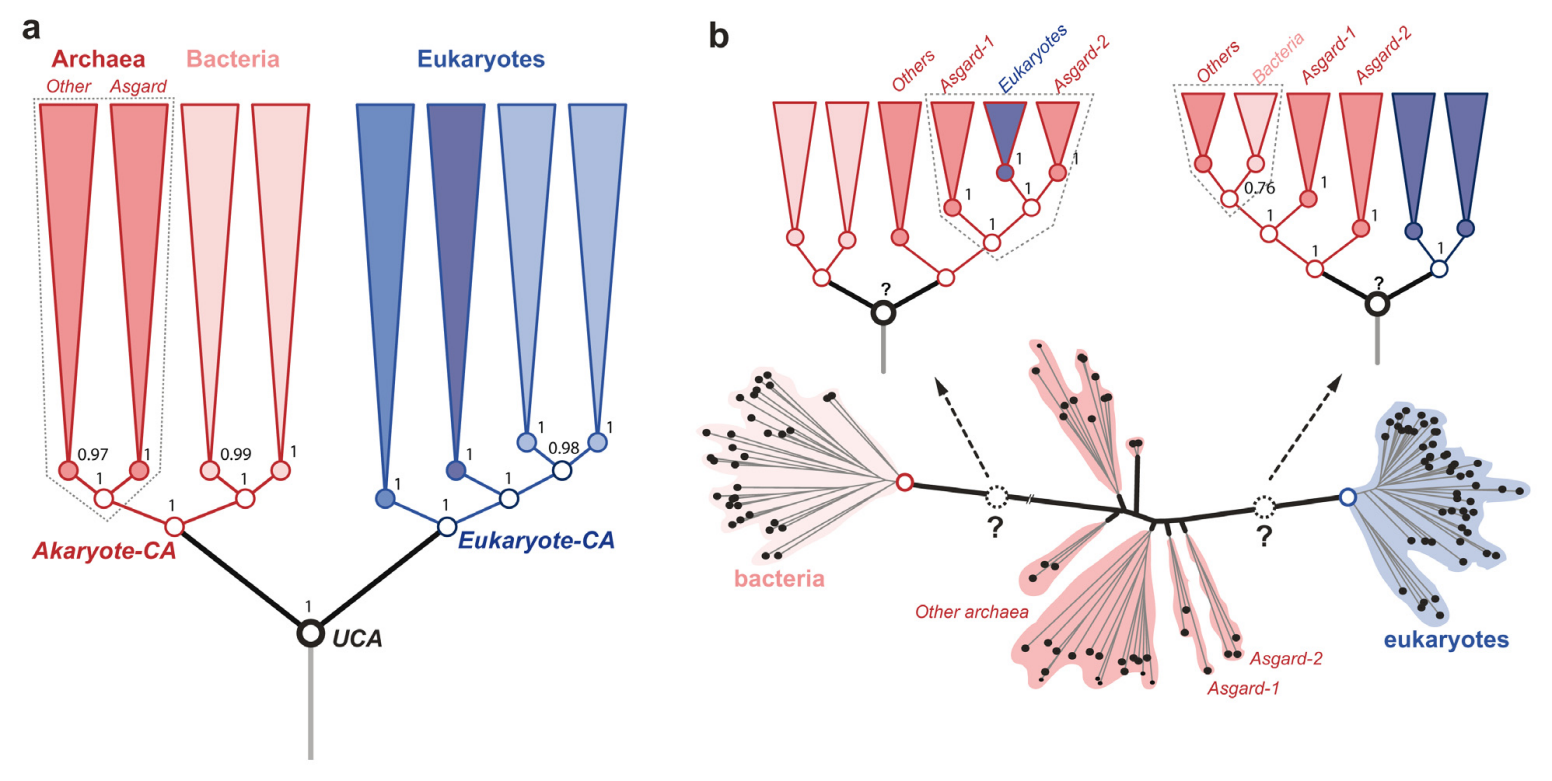
Different 2D “tree of life” (2D-ToL) variants supported by different types of molecular characters using the best-fitting probability models ^1, 4^. ( **a**) The rooted tree (phylogeny) inferred by estimating the evolution of species-specific changes in protein domain composition. Directional character-evolution models place the root between eukaryotes and akaryotes. Named groups of organisms, including Asgardarchaeota are resolved into clades (i.e. a single ancestor). The Asgard archaea are sister to all other archaea, with euryarchaea being the closest relatives. The phylogeny shown is a condensed form obtained after collapsing the clades of the full tree shown previously ^2^. ( **b**) The unrooted tree inferred by estimating the evolution of amino acid composition. The unrooted-tree is the same as in Figure S8d in the article by Williams *et al*. ^7^. The group archaea, and Asgard archaea are unresolved; and a distinct archaeal ancestor is absent. Time-reversible character evolution models cannot identify the root (the universal common ancestor (UCA)) as well. Alternative rootings polarize the branching order in opposite directions implying incompatible relationships among the major organismal clades. Regardless of the rooting, neither Asgard archaea nor archaea as a whole can be resolved as a monophyletic group. Further, Argards do not share a unique common ancestor with other archaea. Even the best-fitting amino acid evolution models cannot resolve the archaeal radiation despite employing thousands of genes ^7^. The poor resolution of archaea is seen in virtually all trees, with or without inclusion of long branches of bacteria. In such ambiguous cases, “character polarization” as in ( **a**) is likely to be efficient, rather than the more commonly used “graphical polarization” of unrooted trees. Clade support is indicated for key groups as ( **a**) Bayesian posterior probability, ( **b**) bootstrap percentage.

The character concept is central to evolutionary biology. Characters are the “data” of evolutionary analyses intended to study evolutionary history and processes of evolution ^1^. Models of character evolution that specify assumptions about the frequency and propensity of character changes are essential for determining the evolutionary relationships of organisms. Phylogenetic analyses based on unique protein-domain characters place Asgardarchaeota (simply Asgards) as sister to other archaea ( Figure 1a), and archaea as sister to bacteria in the tree of life (ToL) ^2– 4^. On the other hand, analyses that employ amino acids as characters fail to resolve the archaeal radiation ( Figure 1b) or to identify a distinct ancestor of archaea ^5– 7^. Conflicts between different reconstructions that employ different character types are often due to incompatible assumptions about character-evolution processes ^8– 10^. In a recent study, Williams *et al*. ^7^ compared the performance of several character-evolution models to evaluate which one of the ToL hypotheses is better supported. The authors tested the performance of different character-evolution models for amino acid characters using empirical data, but models for protein-domain characters with simulated data.

While empirical datasets were limited to at most 1,800 characters, as defined by experimentally determined protein structural-domains ^2, 4, 11, 12^, Williams *et al*. ^7^ generated 1,000,000 simulated characters. They relied on: (i) simulated data to reject a robust phylogeny inferred from empirical data ( Figure 1a) that supports the evolutionary kinship of eukaryotes and akaryotes (the Eukaryote-Akaryote 2D-ToL); and (ii) an assumption consistent with the so-called bacterial rooting to interpret a partially resolved, unrooted-ToL ( Figure 1b), concluding that Asgard archaea are the closest relatives of eukaryotes (the Archaea-Bacteria 2D-ToL) ^7^. Both conclusions are questionable, since: (i) simulated data neither reproduce nor represent empirical distributions, and (ii) poorly resolved trees obscure evolutionary relationships. We argue that Williams *et al*. ^7^ have overlooked important aspects of assessing phylogenetic signal in empirical data, and that it may be premature to reject a well-supported empirical phylogeny ^8– 10^ based on simulated data ^7^.

**Figure 2.**
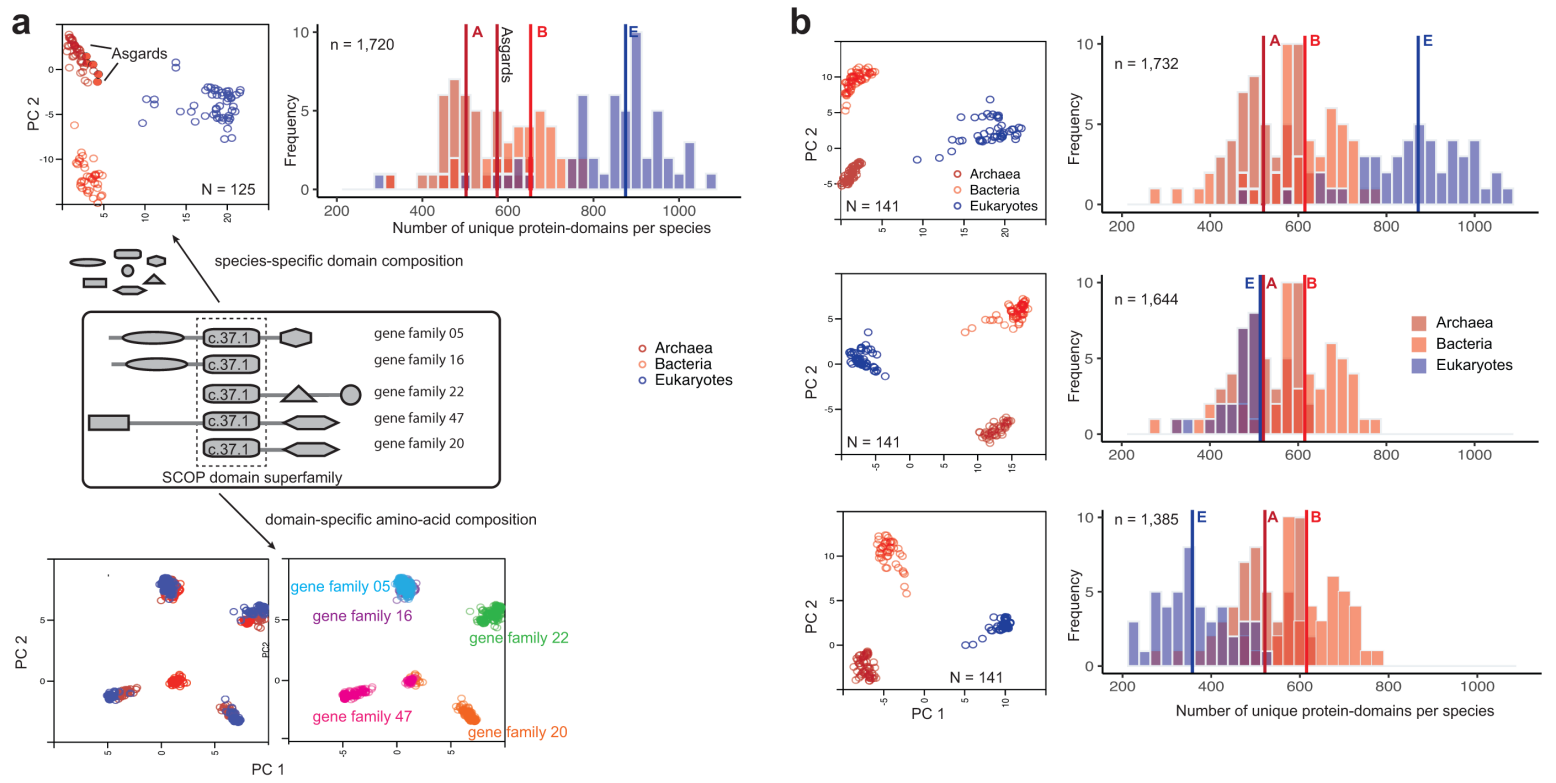
Compositions of unique protein-domains identify with organismal families whereas amino acid compositions of individual domains relate to gene families. ( **a**) Protein-domains are considered to be independent evolutionary units with a distinct tertiary fold, amino acid sequence and biochemical function. A large proportion of proteins are multi-domain proteins formed by duplication and recombination of domain units. Covariation of protein-domain composition among the 125 species sampled by Williams *et al*. ^7^ (top) was compared by principal component analysis (PCA). Each circle in the PCA projection (top left) is a distinct species, defined by a species-specific domain cohort. Asgards are highlighted as filled circles. The frequency distribution (top right) shows the number of distinct protein-domains per species. Vertical intersecting lines in the histograms are the median numbers of protein-domains. Protein domain composition is characteristic of clades of species (top left). In contrast, covariation of amino acid composition (bottom) in a single-domain (super)family is not clade-specific, but instead gene family-specific. Multiple sequence alignments of a single domain (c.37.1) shared by 5/50 concatenated orthologous gene families from 125 species were sampled for the PCA projection. ( **b**) Effects of severe perturbation of the domain composition in recovering clade-specific distributions was tested in a sample of 141 species. Despite the suspicion that the rooting between akaryotes and eukaryotes could be biased due to a larger domain cohort in eukaryotes ^7^, it is not the case ^2, 3, 12^. Diversity of clade-specific domain composition (top right), measured simply as the number of protein domains ^4^, is a poor descriptor of heterogeneity and can be misleading. Clades are grouped by covarying “protein-domain types”, but not by numbers alone. The rooting is stable, and the tree topology is virtually identical, even after reducing the eukaryote cohort by 1/3rds (middle) or 2/3rds (bottom) ^8^ of the original composition ^7^. Descriptions of the PCA projections and frequencies are the same as in ( **a**).

Furthermore, based on simple frequency distributions they suspect that a rooting that separates eukaryotes and akaryotes, as well as the estimates of character compositions of the UCA could be biased. Such simple frequency distributions in extant species can be misleading if they conflate the number of characters with the combinatorics of character compositions ( Figure 2b). Perhaps more importantly, this ignores the historical development of the observed compositions. Indeed, rooting and tree topology are robust against many potential biases ^2– 4, 11^.

Overall, their arguments seem to imply that phylogenies can be inferred only by modeling the evolution of amino acid composition in primary sequence data. We take issue with the view ^7^: “ *However, while protein structure is a useful guide to identifying homology when primary sequence similarity is weak, how best to analyse fold data to resolve deep phylogenetic relationships is still not clear.*” For applications in phylogenomics and systematics, the importance of evaluating molecular homology, and measures to reduce or correct homology errors have been emphasized repeatedly ^9, 13, 14^. Assessment of phylogenies is essentially an assessment of homology, primarily of character homology. Therefore, which 2D-ToL is better supported boils down to: (1) which type of molecular characters and (2) which types of character-evolution models are better for assessing homology.

## Which molecular feature is a better phylogenetic character? Quality over quantity.

Reversibility of amino acid replacements (due to biochemical redundancy) is known to promote convergent/repeated substitutions ^15, 16^. This makes determining character compositions of ancestral nodes ambiguous, as character polarity is ambiguous. This has been a sticking point for locating a distinct archaeal common ancestor (CA), to resolve the phylogeny of the archaeal radiation. This results in a conspicuous absence of the archaeal CA, as well as the universal CA (UCA), in unrooted trees (e.g. Figure 1b), inferred using time-reversible models of character evolution ^5– 7^. Without a distinct node to unite the archaeal branches, the archaea are unresolved, whereas eukaryotes and bacteria are resolved so that their CA nodes are discernable.

Character homology implies a unique historical origin of the character ^2, 17^. The improbability of the repeated/convergent evolution of three-dimensional (3D) structural-domains was demonstrated by an elegant experimental test ^17^. Synthetic versions of a 3D fold were constructed by shuffling the N-C terminal order of segments of the domain to mimic convergent evolution. None of the convergently evolved versions have known homologs. Moreover, complex structural-domains, unlike amino acids, are biochemically non-redundant (see below), and have proven to be excellent molecular characters ^2, 4^ to resolve the deepest branches of the ToL ( Figure 1a). Though undervalued, and underutilized they afford many conceptual and technical advantages over amino acids for phylogenetic modeling ^4, 10, 14^ and estimating ancestral compositions ^3, 4, 12^:

- Substitutions between structural-domains are not known to occur, unlike amino acid replacements, though, domain recombinations that generate new proteins and functions are frequent ^2, 18^. This is because each domain is associated with a distinctive biochemical function.
- There is a natural bias in the propensity for gains and losses, due to physico-chemical constraints on *de novo* generation and convergent evolution of complex domains. This difficulty of parallel gains, and the relative ease of parallel losses, is useful for implementing directional (rooted) character-evolution models ^3, 12, 19^.

A key advantage of using unique characters is that estimating ancestral compositions and evolutionary paths of individual characters is much less ambiguous. In addition to identifying the root nodes, an additional benefit of the built-in directionality is that mutually exclusive evolutionary fates of individual features – inheritance, loss or transfer – can be resolved efficiently using directional-evolution models. For a more thorough discussion of the utility of protein-domains and directional-evolution models to assess homology and non-homology (including horizontal transfer) we refer readers to refs ^2, 11, 12^.

As phylogenetic signal in individual protein-sequence alignments is limited, signal is amplified from multi-protein alignments. The extremely short internode lengths and poor resolution of archaea ( Figure 1b) based on sequence alignments is partly due limited data. That is, they are restricted to at most 10,000 aligned amino acids from 50 proteins, due to the requirement that the aligned genes are present in most/all species under study ^2, 6, 7, 20^. To remedy this, Williams *et al.* ^7^ excluded bacteria, and were able to analyze up to 3,200 protein alignments using coalescent and supertree methods. Both methods do not require all of the aligned proteins to be present in all species sampled, to reconstruct a reconciled/consensus unrooted tree. Williams *et al.* ^7^ claim: (1) a maximally supported clade of eukaryotes and Asgard archaea; and (2) that eukaryotes are the closest relatives of Asgard archaea. However, these conclusions are not possible based on unrooted trees.

To be clear, unrooted trees are not phylogenies per se, since the absence of the root-ancestor(s) obscures ancestor-descendant polarity and phylogenetic relatedness ^14, 15^. Since identifying the closest relatives of extant groups is the same as determining the closeness of their common ancestors, time-reversible models and unrooted trees remain ineffective tools ( Figure 1b). Since the decay of phylogenetic signal in sequence alignments is more pronounced due to repeated substitutions, the uncertainty in estimating ancestral states and locating the deep roots of clades is high.

Furthermore, branch-length estimation from sequences alignments is not a reliable proxy for assessing homology of clades, since it appears to be extremely sensitive to character composition. The latter depends on the inclusion/exclusion of characters, either the choice of: (1) alignable genes, or (2) aligned amino acids (alignment trimming). Both are dependent on the degree of sequence similarity, which can vary wildly in highly divergent taxa and affect the choice of characters. In contrast, the separation of eukaryotes and akaryotes (and of archaea and bacteria) is unperturbed even after extreme perturbation of the domain composition in eukaryotes (e.g. by excluding up to two-thirds of the domain cohort, Figure 2b). The clades within eukaryotes and akaryotes are unperturbed, as well ^11^.

This implies that sequence alignments may not be useful to reliably resolve questions of deep time evolution. Thus, the location of the archaeal-CA or UCA remains ambiguous at best ( Figure 1b), regardless of the gene-aggregation and tree-reconciliation method used for estimating a consensus unrooted tree.

Despite claims to the contrary, that the best-supported root is on the branch separating bacteria and archaea or that eukaryotes are younger than akaryotes ^7^, support from fossils is not reliable either, since assigning fossils to extinct archaea/bacteria or UCA is even more ambiguous. Thus, determining the relative age of eukaryotes and akaryotes requires strong assumptions about the UCA ^7, 21, 22^. Such strong assumptions do not hold when many alternative rootings are tested using protein-domains ^2, 4, 11^. Since estimating ancestral states is much less ambiguous, despite varying species/character sampling and model parameters, rooting between eukaryotes and akaryotes is consistently recovered ( Figure 1a).

## Will more complex models minimize uncertainties or improve phylogenetic signal?

The Eukaryote-Akaryote 2D-ToL reconstructed using parametric rate-heterogenous directional models (e.g. the KVR model) ^19^ is congruent with the ToL inferred from its nonparametric rate-homogenous analog (e.g. the HK model) ^3, 4^. However, Williams *et al.* ^7^ argue that (i) such directional-evolution models may be unsuitable to predict the unique origin of homologous protein-domains along the ToL; and (ii) the Eukaryote-Akaryote 2D-ToL ^8– 10^ is an unsatisfactory explanation of the evolution of the clade-specific compositions of protein domains ( Figure 2).

The KVR model is an extension of the Markov *k* states (Mk) model ^23^, a generic probability model for discrete-state characters. A variant at *k* ≥20 is suitable for modeling evolution of amino acids or copy numbers of gene/protein-domain families. While time-reversible variants produce unrooted trees in which archaea are resolved into a distinct group, such directional models consistently recover a 2D phylogeny in which akaryotes are the closest relatives of eukaryotes ( Figure 1a). The KVR model assumes that the root ancestor has a different character composition from the rest of the tree, which is essentially an irreversible acyclic process. This is fully consistent with the idea that, on a grand scale, the “tree of life” describes broad generalizations of singular events and major transitions underlying striking sister clade differences. Independent/parallel evolution is much less probable for homologous protein-domains or distinct domain permutations (i.e. the specific N-C terminal order of domains), and it is rarely observed compared to amino acid replacements within those domains ^2, 15– 18^. Therefore, the KVR model and its equivalent HK model adequately capture the evolution of complex homologous features, such as 3D protein-domains, if assessing homology is the key criterion.

The assumptions of the KVR model are also consistent with the idea that the idiosyncratic compositions of homologous protein-domains ( Figure 2) is a characteristic of the clades ^2– 4^. In contrast, amino acid compositions in single-domain families are not ( Figure 2a). That is, patterns of covariation of species-specific protein-domain compositions clearly distinguish eukaryotes from akaryotes (and also archaebacteria from eubacteria). The non-random similarity of domain composition within clades, and the systematic covariation of homologous domains among the clades, is referred to as a phylogenetic effect, to imply shared ancestry of the members of a clade. Accordingly, the Akaryote-Eukaryote 2D-ToL ( Figure 1a) was consistently recovered with robust support for the major clades regardless of the taxonomic/protein-domain diversity sampled ( Figure 2b), and regardless of the model complexity ^2– 4, 11, 12^. By contrast, patterns of amino acid covariation are indiscriminant with regard to organismal families, although gene families can be efficiently identified.

Complex variants of the KVR model that account for rate variation among both characters and branches also consistently recovered the Akaryote-Eukaryote 2D-ToL ( Figure 1a), despite significantly different model fits ^2^. More complex models are available, such as the no-common-mechanism model ^24^, an extremely parameter-rich model that allows each character to have its own rate, branch length and topology parameters. Even more complex models can be implemented, which assume that the tempo and mode of evolution changes at each internal node, called node discrete heterogeneity (NDH) models ^7^. However, such over-specified models may not be useful for generalizing the evolutionary process and may over-fit observed patterns – this is a form of model misspecification. For instance, empirical datasets were limited to at most 1,800 domains/characters defined by experimentally determined 3D domains, for phylogenetic analyses using the KVR and HK models. By contrast, Williams *et al*. ^7^ used 1,000,000 simulated characters to estimate the fit between the simulated data and over-complex NDH models.

It is not clear whether the complex over-parameterized models will perform better with empirical datasets. The fact that 1,000,000 characters had to be generated artificially to fit the NDH models suggests that such complex models may not turn out to be efficient, after all. These over-parameterized models are not only likely to be computationally intensive, but are unlikely to be computationally tractable or useful for assessing the homology of unique features, whether molecular or otherwise. This is corroborated by our recent studies in which congruent and virtually identical rooted trees and clades were reconstructed with both parametric rate-heterogeneous models as well as non-parametric rate-homogeneous directional-evolution models ^4, 11^. This congruence is due to the relatively lower heterogeneity of state transition (gain/loss) rates and the compositional heterogeneity of distinct protein-domains (i.e. less noisy data), as compared to the extreme heterogeneity observed in amino acid substitution rates and compositions ^2^. Thus, as mentioned earlier, the relatively simpler KVR/HK models are more than adequate explanations of the empirical datasets. Even if the archaeal radiation remains poorly resolved with more data, the better supported rooting between eukaryotes and akaryotes is consistent with a Eukaryote-Akaryote 2D-ToL ( Figure 1b). That is, diversification of eukaryotes and akaryotes from the UCA is a better supported hypothesis rather than a prokaryote-to-eukaryote transition being assumed to interpret poorly resolved trees.

In conclusion, homology assessment, which is a key to determining relatedness of clades, is a lot simpler and much less ambiguous with complex characters, such as protein-domains, rather than amino acids/nucleotides in sequence alignments ^2, 9, 13^. How best to weight signal from different character types, in order to better resolve different parts of the ToL, is an open question.

## Data and methods

### Data sources

Proteome sequences (predicted protein cohorts from genome sequences) were obtained from recently published studies ^7, 11^. Homologous protein structural domains were identified using the homology assignment tools provided by the SUPERFAMILY database as in previous studies ^2– 4^. Briefly, each proteome was queried against the hidden Markov model (HMM) library of homologous protein-domains defined at the Superfamily level in the SCOP (Structural Classification of Proteins) hierarchy. The taxonomic diversity of sequenced genomes and the number of unique protein domains identified for each species is shown in Table 1.

**Table 1.** Taxonomic diversity and number of unique protein domains assessed.

Study	Number of species sampled per clade	Number of unique protein domains
Williams *et al*. ^7^	125 (Archaea: 39; Bacteria: 33; Eukarya: 52)	1,720
Harish and Kurland ^11^	141 (Archaea: 47; Bacteria: 47; Eukarya: 47)	1,732

### Data analysis

Descriptive statistics of protein-domain compositions for each taxonomic sampling, including the frequency distribution and median number of protein domains for each clade (Archaea, Bacteria and Eukarya), were estimated and visualized using the ggplot2 package (v 3.2.1) in R (v3.6.2). Covariation of clade-specific protein-domain composition, as well as domain-specific amino acid composition, were compared using principal component analysis (PCA). Components were generated by an eigenvector decomposition of the character matrix. PCA scores were based on percentage identity of character compositions.

## Data availability

### Source data

The predicted protein cohorts from genome sequences taken from Williams *et al*. ^7^ and Harish and Kurland ^11^ were assessed.
