# Supplementary material for: The deep(er) roots of Eukaryotes and Akaryotes
Source: F1000Res. 2020 Jun 22;9:112. Originally published 2020 Feb 13. [Version 2] doi: 10.12688/f1000research.22338.2 (PMC7336049; doi:10.12688/f1000research.22338.2)
Supplement: Peer review history

# Peer review: Reviewer response for version 2

**DOI:** 10.5256/f1000research.27407.r65208

**Published:** 2020-07-03

**Authors:** John Gatesy

**Affiliations:** 1 Division of Vertebrate Zoology, Sackler Institute for Comparative Genomics, American Museum of Natural History, New York City, NY, USA

Are arguments sufficiently supported by evidence from the published literature or by new data and results?

Partly

Is the conclusion balanced and justified on the basis of the presented arguments?

Partly

Is the rationale for commenting on the previous publication clearly described?

Yes

Are any opinions stated well-argued, clear and cogent?

Partly

Reviewer Expertise:

NA

I confirm that I have read this submission and believe that I have an appropriate level of expertise to confirm that it is of an acceptable scientific standard.

# Peer review: Reviewer response for version 1

**DOI:** 10.5256/f1000research.24642.r63265

**Published:** 2020-05-29

**Authors:** John Gatesy

**Affiliations:** 1 Division of Vertebrate Zoology, Sackler Institute for Comparative Genomics, American Museum of Natural History, New York City, NY, USA

Harish and Morrison explore rooting the tree of Life given recently proposed hypotheses. They might consider the following in editing/improving their manuscript:

  

1. First sentence of the background. Not sure I agree; it depends on what the authors think a 'model' is in this context. Are they referring to an explicit substitution or transition rate matrix model, or some more general concept?
2. Definition of 'domains' would seem to be more ambiguous than defining particular amino acids that are discrete subunits with a very simple genetic basis. Admittedly, the alignment of such amino acids can entail ambiguity, especially at these divergences, so I suppose all inference at this level is a challenge. But, determining whether a particular 'domain' is even a 'domain' (or not two domains or one and a half domains or a different domain) is in my view squishy. 
3. Rooting using asymmetrical abstractions (models) is squishy too. This is basically never done except for when there is no outgroup. In this case, there is none, but I am disturbed by the authors confidence (e.g., Fig. 1a with maximum support) in rooting an ancient tree based on this model or that.
4. Page 4 left column. I think that the following is an assertion, not a fact, "Substitutions between structural domains do not occur, unlike amino acid replacements, since each domain defines a distinctive biochemical function". Who says that one domain cannot transform into another? I do not understand this assertion. The authors have extreme confidence in this statement it seems, but perhaps this is the problem? Were they there, back 100s of millions of years ago to observe that one domain could not have evolved into another or that similar domains did not evolve convergently into what the authors assume are the same domain (even though it might not be the same 'domain')?
5. Page 4 left column. I am assuming that the authors' preferred models, "The natural bias in gain/loss rates, arising from the difficulty of parallel gains and the relative ease of parallel losses, is useful for implementing directional (rooted) character-evolution models". The idea that there is some general rate across all domains for gain and loss and convergent gain seems naive to me, or at least, a poor criterion for rooting a tree with awesome confidence and high probability.
6. I am not buying the idea that these domains are 'non-redundant'. I believe that the authors believe this, but that is about it. So, I do not think there is "built in directionality" if the authors' initial assumptions/assertions are accepted. It is true that one can root a tree if one assumes one can identify homologous domains accurately and apply a very specific general model to a situation that is not specific and surely not general in terms of rate. Rooting the tree of Life will always be depedent on some sort of model that assumes this or that about gains and losses and convergent gains as there is no outgroup (whether gains or losses of domains or genes or nucleotides or amino acids), but this just reinforces the authors' initial assertion in the paper that models that people imagine (which are poorly understood in terms of process) will drive results. The fact that the amino acid trees (unrooted) are completely in conflict with the domain-based tree is not a good sign as no congruence among different data, even in an unrooted context. I suppose it is okay for the authors to assert their tree is better, but I think many people will not agree or be convinced by trust in some general asymmetry model and domains that may or may not be the same thing in very divergent taxa. From the amino acid analysis side of the debate, their tree seems to refute the domain tree, even though it is unrooted (and vice versa I guess), so as an outside viewer of this debate, there seems to be a lot of work to do on an admittedly challenging problem. But, that was likely known before this contribution.
7. Do the authors' asymmetry models take lateral transfer of domains into account as well? Since the authors admit that, "Further, such incompatibilities are likely to make estimating the absolute origins of single-domain families and single genes difficult, since a majority of genes are formed by duplication and recombination of distinct domains", if their asymmetry domain models for rooting do not take lateral transfer into account, this would seem problematic to me, given that they argue for the importance of lateral transfer of entire genes (and genes include 'domains').
8. I think the following from the authors is an assertion, not a fact, "Since parallel evolution of homologous protein-domains or distinct domain permutations is very rare, the KVR model adequately captures the evolution of unique features." If not a necessarily true, nothing the authors argue is either? 
9. The authors note that, "The systematic covariation of homologous domains among the clades is best explained as phylogenetic effect". I have studied phylogenetics for 30 years, yet I still have not seen any compelling or useful definition of this term that makes any sense. This just seems like a vague explanation for an observed pattern of covariation. Many of the things that the authors seem to consider 'clade-specific' could just be 'grade-specific'. For example, 'fish' have lots of common features that sort of make sense together as all primitively swim around in water, have tails for propulsion underwater, and breath oxygen and feed underwater. Similarly, 'domains' characteristic of eukaryotes or archaeans might not define monophyletic groups, but might instead be characteristic of paraphyletic groups (e.g., Asgards or 'Others' in Fig. 1b). So, one wonders whether the robustly rooted tree of Life based on specifics of a particular model mean that much, or not. The fact that the tree strongly contradicts an unrooted tree based on independent data (Fig. 1b) does not give me much confidence as an outsider to the debate.
10. This has to be a gross overstatement? "The KVR model is an optimal explanation of the evolution of clade-specific composition of homologous features." For example, the authors note that, "More complex models are available, such as the no- common-mechanism model, an extremely parameter-rich model that allows each character to have its own rate, branch length and topology parameters." For this model, surely there would be fewer evolutionary steps; wouldn't that be more 'optimal'? What is the result for this model that could be interpreted as a better (more optimal?) fit to the data - certainly not optimal in terms of minimizing evolutionary steps, unless the tree topology for the model based analysis and parsimony are identical.

Are arguments sufficiently supported by evidence from the published literature or by new data and results?

Partly

Is the conclusion balanced and justified on the basis of the presented arguments?

Partly

Is the rationale for commenting on the previous publication clearly described?

Yes

Are any opinions stated well-argued, clear and cogent?

Partly

Reviewer Expertise:

phylogenetics

I confirm that I have read this submission and believe that I have an appropriate level of expertise to state that I do not consider it to be of an acceptable scientific standard, for reasons outlined above.

# Response

**Published:** 2020-06-15

**Authors:** Ajith Harish

**Affiliations:** Unaffiliated, Sweden

**Response to reviewer**

We thank the reviewer for their suggestions. The comments helped us improve the clarity of the presentation. We revised the text extensively to address the issues raised.

**Suggestion:**

1.     First sentence of the background. Not sure I agree; it depends on what the authors think a 'model' is in this context. Are they referring to an explicit substitution or transition rate matrix model, or some more general concept?

**Response:** We revised it as: “Models of character evolution that specify assumptions about the frequency and propensity of character changes”

**Suggestion:** 

2.     Definition of 'domains' would seem to be more ambiguous than defining particular amino acids that are discrete subunits with a very simple genetic basis. Admittedly, the alignment of such amino acids can entail ambiguity, especially at these divergences, so I suppose all inference at this level is a challenge. But, determining whether a particular 'domain' is even a 'domain' (or not two domains or one and a half domains or a different domain) is in my view squishy. 

**Response: **We use domain definitions from experimentally determined structures according to the SCOP (Structural Classification of Protein) scheme. In general, an independently folding unit is considered to be a domain. There is a large body of experimental work and literature on delimiting domains based on 3D structure and function as well as assigning domains and determining homology using computational tools. Arguably, assessing homology of a domain (or a protein) is more reliable than determining the homology of amino acids/nucleotides even in the absence of alignment ambiguities.

**Suggestion:**

3.     Rooting using asymmetrical abstractions (models) is squishy too. This is basically never done except for when there is no outgroup. In this case, there is none, but I am disturbed by the authors confidence (e.g., Fig. 1a with maximum support) in rooting an ancient tree based on this model or that.

**Response: **Our confidence and maximum support are based on the consistency of the rooting in all sampled “rooted trees” (> 50,000 after burnin). In addition, the reason for our confidence is that the alternative rootings have negligible support based on Bayes Factor estimates as shown in our earlier studies (Refs 2, 4 & 11).

Agreed that most phylogeny software are designed to output/read unrooted trees, and so the support value for the root split is not usually reported, because it is not calculated.

**Suggestion:**

4.     Page 4 left column. I think that the following is an assertion, not a fact, "Substitutions between structural domains do not occur, unlike amino acid replacements, since each domain defines a distinctive biochemical function". Who says that one domain cannot transform into another? I do not understand this assertion. The authors have extreme confidence in this statement it seems, but perhaps this is the problem? Were they there, back 100s of millions of years ago to observe that one domain could not have evolved into another or that similar domains did not evolve convergently into what the authors assume are the same domain (even though it might not be the same 'domain')?

**Response: **Substitutions between structural-domains are not known to occur, unlike amino acid replacements, though, domain recombinations that generate new proteins and functions are frequent ^2,18^. This is because each domain is associated with a distinctive biochemical function.

We also included a reference (Bashton, M. & Chothia, C. The Generation of New Protein Functions by the Combination of Domains. Structure 15, 85–99 (2007).). We hope this will be useful to the readers as to why substitutions of domains are not known, or why they may not be possible. It could be useful to answer the questions raised in the previous comment.

**Suggestion:**

5.     Page 4 left column. I am assuming that the authors' preferred models, "The natural bias in gain/loss rates, arising from the difficulty of parallel gains and the relative ease of parallel losses, is useful for implementing directional (rooted) character-evolution models". The idea that there is some general rate across all domains for gain and loss and convergent gain seems naive to me, or at least, a poor criterion for rooting a tree with awesome confidence and high probability.

6.     I am not buying the idea that these domains are 'non-redundant'. I believe that the authors believe this, but that is about it. So, I do not think there is "built in directionality" if the authors' initial assumptions/assertions are accepted. It is true that one can root a tree if one assumes one can identify homologous domains accurately and apply a very specific general model to a situation that is not specific and surely not general in terms of rate. Rooting the tree of Life will always be depedent on some sort of model that assumes this or that about gains and losses and convergent gains as there is no outgroup (whether gains or losses of domains or genes or nucleotides or amino acids), but this just reinforces the authors' initial assertion in the paper that models that people imagine (which are poorly understood in terms of process) will drive results. The fact that the amino acid trees (unrooted) are completely in conflict with the domain-based tree is not a good sign as no congruence among different data, even in an unrooted context. I suppose it is okay for the authors to assert their tree is better, but I think many people will not agree or be convinced by trust in some general asymmetry model and domains that may or may not be the same thing in very divergent taxa. From the amino acid analysis side of the debate, their tree seems to refute the domain tree, even though it is unrooted (and vice versa I guess), so as an outside viewer of this debate, there seems to be a lot of work to do on an admittedly challenging problem. But, that was likely known before this contribution.

7.     Do the authors' asymmetry models take lateral transfer of domains into account as well? Since the authors admit that, "Further, such incompatibilities are likely to make estimating the absolute origins of single-domain families and single genes difficult, since a majority of genes are formed by duplication and recombination of distinct domains", if their asymmetry domain models for rooting do not take lateral transfer into account, this would seem problematic to me, given that they argue for the importance of lateral transfer of entire genes (and genes include 'domains').

8.     I think the following from the authors is an assertion, not a fact, "Since parallel evolution of homologous protein-domains or distinct domain permutations is very rare, the KVR model adequately captures the evolution of unique features." If not a necessarily true, nothing the authors argue is either? 

**Response: **We edited the text and re-wrote parts of the text to address issues raised in points 5-8. For a more detailed discussion of these matters, we recommend references 2-4, in addition to the ones mentioned below. But in brief,

(a) The directional models do not assume a general rate of gain/loss. The relative rates were estimated using a Gamma distribution, up to 12 rate categories, and did not affect the rooting or tree topology (see refs 2, 4).

(b) Convergent evolution of the same 3D structure is highly improbable given the what we know about the sequence-structure relationships. We have now included a reference that shows the improbability of the convergent evolution with an elegant experiment (Mackin, K. A., Roy, R. A. & Theobald, D. L. An empirical test of convergent evolution in rhodopsins. Mol. Biol. Evol. 31, 85–95 (2014)).

(c) The skewed rates of losses over gains of unique gene families have been reported in other studies using presence/absence of genes (e.g. Zamani-Dahaj SA, Okasha M, Kosakowski J, Higgs PG. Estimating the frequency of horizontal gene transfer using phylogenetic models of gene gain and loss. Molecular biology and evolution. 2016 Jul 1;33(7):1843-57.).

(d) In practice, it is not easy to distinguish convergent evolution from horizontal transfer (HT) using presence/absence patterns by itself, but given (b) and (c), HTs are a minority.

**Suggestion:**

9.     The authors note that, "The systematic covariation of homologous domains among the clades is best explained as phylogenetic effect". I have studied phylogenetics for 30 years, yet I still have not seen any compelling or useful definition of this term that makes any sense. This just seems like a vague explanation for an observed pattern of covariation. Many of the things that the authors seem to consider 'clade-specific' could just be 'grade-specific'. For example, 'fish' have lots of common features that sort of make sense together as all primitively swim around in water, have tails for propulsion underwater, and breath oxygen and feed underwater. Similarly, 'domains' characteristic of eukaryotes or archaeans might not define monophyletic groups, but might instead be characteristic of paraphyletic groups (e.g., Asgards or 'Others' in Fig. 1b). So, one wonders whether the robustly rooted tree of Life based on specifics of a particular model mean that much, or not. The fact that the tree strongly contradicts an unrooted tree based on independent data (Fig. 1b) does not give me much confidence as an outsider to the debate.

**Response: **We revised the sentence as “The non-random similarity of domain composition within clades and the systematic covariation of homologous domains among the clades is referred to as phylogenetic effect to imply shared ancestry of the members of a clade”. Moreover, as we have now clarified, we hope we can agree that homology assessment is key to assessing phylogenies. If one agrees that protein domains are better characters to assess homology than nucleotides/amino acids, then the phylogenies estimated with protein domains are indeed better to assess the relatedness of eukaryotes and akaryotes.

**Suggestion:**

10.  This has to be a gross overstatement? "The KVR model is an optimal explanation of the evolution of clade-specific composition of homologous features." For example, the authors note that, "More complex models are available, such as the no- common-mechanism model, an extremely parameter-rich model that allows each character to have its own rate, branch length and topology parameters." For this model, surely there would be fewer evolutionary steps; wouldn't that be more 'optimal'? What is the result for this model that could be interpreted as a better (more optimal?) fit to the data - certainly not optimal in terms of minimizing evolutionary steps, unless the tree topology for the model based analysis and parsimony are identical.

**Response: **We revised this statement and expanded the discussion in the penultimate paragraph of section 2. We clarify why the relatively simpler directional-evolution models such as the parametric KVR model and its non-parametric (parsimony) analog HK model are adequate for empirical data (1,800 characters) as opposed to the 1,000,000 simulated characters required to estimate the fit of data to the more complex models. The KVR and HK models do recover congruent phylogenies.

# Peer review: Reviewer response for version 1

**DOI:** 10.5256/f1000research.24642.r60012

**Published:** 2020-05-04

**Authors:** Jacob S. Berv, Stephen A. Smith

**Affiliations:** 1 Department of Ecology and Evolutionary Biology and Museum of Paleontology, University of Michigan, Ann Arbor, MI, USA; 2 Department of Ecology and Evolutionary Biology, University of Michigan, Ann Arbor, MI, USA

**Review of: The deep(er) roots of Eukaryotes and Akaryotes**

 Jacob S. Berv and Stephen A. Smith

**Introduction**

 In the present article (Harish and Morrison, 2020), Harish and Morrison argue that prior work (Williams *et al.*, 2020 ^1^) to elucidate the structure of the deepest branches of the tree of life, was misled by reliance on particular data types and models which are unsuited to the task. In general, we agree that Harish and Morrison has merit as a scientific contribution, and represents a valid perspective. However, we are nonetheless cautious as to their conclusions.

 The debate at issue concerns several hypotheses concerning the monophyly and placement of archaea, bacteria, and eukaryotes. Williams *et al.*’s rigorous phylogenetic analysis of “redundant” characters, like nucleotide or amino acid sequences, converges on a solution that places eukaryotes within the archaea, and as sister to bacteria – in a two-domain tree of life. This paper garnered significant attention at the time of publication earlier this year, and given the implications of their result, it is fair to subject it to additional scrutiny. The present article by Harish and Morrison, therefore, constitutes a rebuttal to Williams *et al.*, which itself is in part a response to earlier work by Harish *et al. *(Harish, 2018; Harish and Kurland, 2017a, 2017b, 2017c) ^2^^,^^3^^,^^4^^,^^5^.

 Before we can have confidence in understanding the branching pattern reflecting at the root of the tree of life, it seems important to acknowledge that there are several key questions at play that are frequently confounded, as rightly emphasized by this and prior work by Harish *et al.*: 1) What is a domain? 2) How many domains are there? 3) What are the relationships of these domains to each other? Clearly articulating and answering these questions will require addressing two issues that have plagued prior studies of the origins of crown-group life. The first involves the information content of particular data types and identifying which data types are most likely to contain information relevant for discriminating between particular phylogenetic hypotheses. There is significant literature on this front, both from theoretical and empirical perspectives (e.g Dornburg *et al*., 2019; Reddy *et al.*, 2017; Townsend and Leuenberger, 2011 ^6^^,^^7^^,^^8^). The second key issue concerns the rooting of the tree of life, which presents several difficult challenges that may require addressing fundamental epistemological choices (below). In our review here, we will briefly outline these two issues and discuss the arguments presented by the article by Harish and Morrison.

**Information content**

 Harish and Morrison argue for a two-domain tree of life that places a clade of archaea and bacteria (together called Akaryotes) as sister to Eukaryotes. In particular, Harish and Morrison argue that phylogenetic characters derived from the presence/absence of protein domains are more suited to the task of elucidating the deepest roots of the tree of life than more traditional phylogenetic characters advocated for by Williams *et al*. Protein structural domains, which are ~200 amino acid or ~600 nucleotides long, each with unique structure and function (Harish, 2018), have been the focus of prior work by the authors, and we find the authors’ arguments in favor of their application to be justifiable. These data types, at the very least, serve as complementary to other data types used for phylogenetic reconstruction and offer some compelling properties relevant for deep phylogenetic reconstruction.

 Harish and Morrison point out that unlike traditional nucleotides and amino acid characters, structural domains may be relatively homoplasy free and therefore useful for clarifying the extremely difficult problem of the root of the tree of life. Using such characters for phylogenetic inference recognizes homology of structure that may be lost at the sequence level. Structural domains also exhibit compositional variance that isolates species into taxonomic clusters, whereas clustering of amino acid data generates clusters that reflect gene families, and not clades (Figure 2a). While there may be lineage specific compositional heterogeneity of amino acids within gene families, models that assume amino acid composition to be consistent across gene regions may be a poor fit to data.

 One issue not addressed by Harish and Morrison but that we feel warrants comment regards branch lengths. A qualitative comparison of the branch lengths reported from prior work by Harish *et al.*, (relying on protein domain characters), and the branch lengths reported in Williams *et al.* (relying on amino acid sequences), is strongly suggestive of the hypothesis that the trees reported in Williams *et al. *may be influenced by saturation artifacts. In Figure 2a and of Williams *et al*, the branch lengths connecting bacteria and eukaryotes to their respective positions in the unrooted tree of life are much greater than 1 substitution per site. This suggests that there are few to no sites in the alignment that are not variable at edges separating major putative clusters in these data. In other words, there may be no detectable homology between these clades and the rest of the data. This is a very high rate of evolutionary change in the context of divergences that occurred billions of years ago. Williams *et al. *discuss that the reason these branches may be so long is that the CAT+GTR+G4 model is better able to identify convergent substitutions on these branches (as validated by posterior predictive simulation). While this may be true, an alternative interpretation is that there is simply no information in those amino acid data directly relevant to this question, and different models are reflecting statistical differences, and not necessarily different signals in the data. In comparison, the branch lengths reported by Harish (2018) (for instance) seem to indicate much more realistic values (Figure 6 in Harish, 2018) indicates all branches are much shorter than 1 substitution/site). These observations, at the very least, suggest caution in interpreting the Williams *et al. *result without further analysis of the adequacy of the model to fit these data with so few shared characters, and would seem to argue in favor of more slowly evolving (but nonetheless apparently very informative) characters like protein domains employed by Harish *et al*. The rate of evolution across characters is an important consideration in addition to the rate of evolution of the locus when examining the utility of a particular data type for resolving a phylogenetic question (Dornburg et al., 2019).

 While the branch lengths of Williams *et al. *may give one pause, some relationships in 

 Harish and Kurland (2017b) also warrant discussion. The rooted analyses that place Eukaryotes sister to Akaryotes result in several relationships that would be considered unusual as compared to most other analyses that focus on the resolution of early Eukaryotes. For example, the resulting analyses have plants as paraphyletic with strong support. Other relationships, while perhaps less egregiously different than other analyses are still uncommon. These results might call into question the quality of these data and analyses for resolving other relationships.

**Rooting**

 Harish and Morrison argue that “non-redundant” protein domain characters provide a key advantage which allows them to aid in directly estimating the position of the root of the tree of life, using non-reversible models. The issue of rooting cannot be overstated, and its lack of consideration by Williams *et al.* is an oversight. An unrooted tree does not describe phylogenetic relationships including monophyly. While typical phylogenetic analyses include an outgroup on which the tree can be rooted, the root of the tree of life presents distinct challenges. Harish (2018) discusses why rooting the tree of life is perhaps the most difficult phylogenetic problem: in the absence of outgroups or fossils, the typical approach has been to root the tree on bacteria, but of course, “the nearest neighbor in an unrooted tree need not necessarily be the closest relative” Harish (2018). We reiterate these sentiments – identifying support for the existence of a particular monophyletic clade does not constitute evidence of its relationships with other monophyletic groups—indeed, with an unknown root position, many alternative topological optimizations can be generated by attaching the root to different branches among the three domains a posteriori, which each fundamentally altering our understanding of the origins of crown-group life.

 The common practice of rooting the ToL with bacteria is unsatisfying as it enforces a strong assumption, and we, therefore, agree with Harish and Morrison that directional models of character evolution may be a useful way forward. The KVR model (Klopfstein *et al.*, 2015) ^9^ Haris and Morrison advocate for assumes that the root possesses a different character composition than descendants, and since independent convergence of protein domains may be very rare, the KVR model may be informative in optimizing the position of an unknown root. Williams *et al. *investigated this proposal by Harish *et al. *through simulations and found that with simulated datasets that allowed protein fold compositions to vary over the tree, the KVR model often fails to find the correct root. They note that the root position appears to systematically converge toward branches that represent a majority of the compositional variance, which can be controlled by including or excluding taxa from within subclades. Harish and Morrison argue that the simulations employed by Williams *et al. *do not accurately capture important features of the empirical data, and so are of limited relevance. They note that their own experiments, which reduce the eukaryote sample by ½ or ⅔ still recover a stable root position between Akaryotes and Eukaryotes. These perspectives suggest a strong disagreement over what may be the most critical aspect of a larger problem. It seems to us therefore that continued development of approaches to objectively identify the position of the root may be helpful in making progress, whether or not the topology advocated for by Harish and Morrison is correct.

 In sum, the present work by Harish and Morrison serves to emphasize that there are still a number of unresolved challenges in understanding the deepest roots of the tree of life. The development of tools like posterior predictive simulation may help us understand how well our models capture specific aspects of our data (e.g. Brown, 2014; Foster, 2004) ^10^^,^^11^, but it will also be important to consider the likely signal of homology that may be present in different data types, as well as how best to objectively identify the position of the root of the tree of life.

Are arguments sufficiently supported by evidence from the published literature or by new data and results?

Partly

Is the conclusion balanced and justified on the basis of the presented arguments?

Yes

Is the rationale for commenting on the previous publication clearly described?

Yes

Are any opinions stated well-argued, clear and cogent?

Yes

Reviewer Expertise:

molecular systematics and phylogenetics

We confirm that we have read this submission and believe that we have an appropriate level of expertise to confirm that it is of an acceptable scientific standard, however we have significant reservations, as outlined above.

## References

[1] : Phylogenomics provides robust support for a two-domains tree of life. *Nat Ecol Evol*.4(1) : 10.1038/s41559-019-1040-x 138-147 10.1038/s41559-019-1040-x 31819234PMC6942926

[2] : What is an archaeon and are the Archaea really unique?. *PeerJ*.2018;6: 10.7717/peerj.5770 e5770 10.7717/peerj.5770 30357005PMC6196074

[3] : Mitochondria are not captive bacteria. *Journal of Theoretical Biology*.2017;434: 10.1016/j.jtbi.2017.07.011 88-98 10.1016/j.jtbi.2017.07.011 28754286

[4] : Akaryotes and Eukaryotes are independent descendants of a universal common ancestor. *Biochimie*.2017;138: 10.1016/j.biochi.2017.04.013 168-183 10.1016/j.biochi.2017.04.013 28461155

[5] : Empirical genome evolution models root the tree of life. *Biochimie*.2017;138: 10.1016/j.biochi.2017.04.014 137-155 10.1016/j.biochi.2017.04.014 28478110

[6] : Optimal Rates for Phylogenetic Inference and Experimental Design in the Era of Genome-Scale Data Sets. *Systematic Biology*.2019;68(1) : 10.1093/sysbio/syy047 145-156 10.1093/sysbio/syy047 29939341

[7] : Why Do Phylogenomic Data Sets Yield Conflicting Trees? Data Type Influences the Avian Tree of Life more than Taxon Sampling. *Syst Biol*.2017;66(5) : 10.1093/sysbio/syx041 857-879 10.1093/sysbio/syx041 28369655

[8] : Taxon sampling and the optimal rates of evolution for phylogenetic inference. *Syst Biol*.2011;60(3) : 10.1093/sysbio/syq097 358-65 10.1093/sysbio/syq097 21303824

[9] : A Nonstationary Markov Model Detects Directional Evolution in Hymenopteran Morphology. *Syst Biol*.2015;64(6) : 10.1093/sysbio/syv052 1089-103 10.1093/sysbio/syv052 26272507PMC4604834

[10] : Detection of implausible phylogenetic inferences using posterior predictive assessment of model fit. *Syst Biol*.2014;63(3) : 10.1093/sysbio/syu002 334-48 10.1093/sysbio/syu002 24415681

[11] : Modeling compositional heterogeneity. *Syst Biol*.2004;53(3) : 10.1080/10635150490445779 485-95 10.1080/10635150490445779 15503675

# Response

**Published:** 2020-06-15

**Authors:** Ajith Harish

**Affiliations:** Unaffiliated, Sweden

**Response to reviewers**

We thank the reviewers for their detailed review and suggestions.

**​​​​​​​**

**Suggestion:** One issue not addressed by Harish and Morrison but that we feel warrants comment regards branch lengths.

**Response:** We have now included a discussion of branch lengths, and how branch lengths are not reliable proxies for assessing homology of clades, in the second to last paragraph of section 1, as follows: “Furthermore, branch-length estimation from sequences alignments is not a reliable proxy for assessing homology of clades, since it appears to be extremely sensitive to character composition. The latter depends on the inclusion/exclusion of characters, either the choice of: (1) alignable genes, or (2) aligned amino acids (alignment trimming). Both are dependent on the degree of sequence similarity, which can vary wildly in highly divergent taxa and affect the choice of characters. In contrast, the separation of eukaryotes and akaryotes (and of archaea and bacteria) is unperturbed even after extreme perturbation of the domain composition in eukaryotes (e.g. by excluding up to two-thirds of the domain cohort, Figure 2b). The clades within eukaryotes and akaryotes are unperturbed as well ^11^.”.

# Peer review: Reviewer response for version 1

**DOI:** 10.5256/f1000research.24642.r60618

**Published:** 2020-03-31

**Authors:** Edward Braun

**Affiliations:** 1 Department of Biology, Genetics Institute, University of Florida, Gainesville, FL, USA

It is challenging to review a reply without considering the original paper carefully. In this case it is doubly challenging because the most relevant portion of Williams *et al.* (2020) ^1^ is itself a reply to Harish and Kurland (2017) ^2^. This prompted me reread Harish and Kurland (2017) and Williams *et al.* (2020) in detail and evaluate the Harish and Morrison manuscript (which I will call HM-F1000 hereafter) in light of those publications. Thus, I have decided to provide a review of HM-F1000 along with a limited post-publication review of Harish and Kurland (2017) and Williams *et al.* (2020). I will divide this review into two sections: 1) a discussion of the larger philosophical questions and 2) a description of the changes to HM-F1000 that I believe to be necessary as well as a few minor issues with HM-F1000.

 Before I provide that combined review, I want to answer two questions: 1) does HM-F1000 warrant publication as a peer-reviewed publication? and 2) did HM-F1000 convince me that the Eukaryote/Akaryote* two-domain tree of life (2D-ToL) represents an accurate placement of the root of the tree of life (ToL)? My answer to the first question is “ yes” and my answer to the second question is “ not at this time.”

 * NOTE: Throughout this review I will use “akaryote” in because it is the terminology used in HM-F1000; however, I am agnostic regarding benefits of that term relative to “prokaryote.”

 I have answered the first question in the affirmative despite expressing substantially greater caution when I answer the second because identifying the position for the root of the ToL is arguably one of the most difficult problems in evolutionary biology. The field needs more ideas regarding the best way to estimate a robust topology for the ToL and place the root, not fewer. Excluding the HM-F1000 from the peer-reviewed literature would exclude their concise defense of the idea that the KVR (Klopfstein *et al.* 2015) ^3^ model of evolution can be used with protein fold presence/absence data to place the root of the ToL.

 *****

**Section 1:**

 Williams *et al.* (2020) argued that using the KVR model with protein domain composition data was inappropriate based upon their simulations; they chose instead to focus on analyses using models of protein sequence evolution. It is certainly true that the KVR model is a simplistic model of evolution. However, the fact that the KVR model is imperfect does not invalidate the use of the model; as Box (1979) famously stated “all models are wrong but some are useful.”

 Simply showing that that a model has imperfect fit to empirical data (as Williams *et al.* 2020 did) does not mean the model is useless for inference. Indeed, it is very likely that the models of protein evolution, like the CAT, C60, and NDCH2 models, which Williams *et al. *(2020) used in their other analyses, also have an imperfect fit to the true underlying process of evolution. The central issue is whether analyses of protein fold data using the KVR model are more likely to recover true historical signal than analyses of aligned proteins using various models of protein sequence evolution.

 HM-F1000 highlights a corollary of this fundamental issue in its abstract when they state that “(i)t is well known that different character types present different perspectives on evolutionary history that relate to different phylogenetic depths.” With that said, is clear that the models of protein sequence evolution that Williams *et al.* (2020) used are much more sophisticated than the KVR model. So why should we embrace the results of the KVR model over the results of analyses using those sophisticated models of protein evolution? Obviously, the purpose of HM-F1000 is to convince readers to accept the results of the KVR model (applied to protein fold presence/absence data) as more likely to be correct (in this context I will use “correct” to mean “closer to the truth”) than the results of the models of protein evolution used by Williams *et al.* (2020). Why might that be the case? I can think of two reasons that I will discuss below:

 ---

 I. Historical signal might decay more rapidly in aligned protein sequences than in protein fold content data.

 The simplest explanation for preferring the results of analyses using the protein fold data to those obtained using aligned proteins is the possibility that historical signal might have decayed in the latter. Mossel (2003) ^4^ proved that “…it is impossible to reconstruct the topology of ‘deep’ trees with high mutation rates…” More accurately, Mossel (2003) identified a bound on the number of characters necessary for tree reconstruction, but that bound implies that impossible to reconstruct some past events (also see Sober and Steel 2002) ^5^. Perhaps protein sequence alignments cannot provide accurate information about the deepest branches in the tree of life and we have to look to other data types, like protein fold content, to estimate the topology of the deepest branches in the tree of life.

 In my opinion, two arguments are necessary to establish that data type is more important that model fit for reconstructing the deepest branches in the tree of life. HM-F1000 states that “(p)rotein structural domains, unlike amino acids, are biochemically non-redundant (see below) and have proven to be excellent ‘genomic characters’…” as a defense of the idea that protein fold data might be superior to aligned amino acids. I was expecting an explicit statement that it might be appropriate to view changes in the protein fold repertoire as rare genomic changes (RGCs; Rokas and Holland 2000; Bleidorn 2017) ^6^^,^^7^. I think that linking protein folds to RGCs is important because it provides an explicit link between protein fold data and the body of theory surrounding RGCs. Specifically, the fact that analyses using the maximum parsimony criterion are expected yield the correct tree when applied to RGC data (Steel and Penny 2004; 2005) ^8^^,^^9^. I believe this has implications for the idea that the relatively simple KVR model might be useful for rooting the tree of life.

 Whether the maximum parsimony criterion should be viewed as a simple model (or any sort of model) has been a topic of philosophical debate in phylogenetics (Goloboff 2003’ Huelsenbeck *et al.* 2008) ^10^^,^^11^; I will accept the idea that maximum parsimony is “simple” for the sake of this argument (also see Yang 1995) ^12^. However, if we accept that a “perfect RGC” model (which I define as a process that results in some binary character that can only undergo a single transition on one edge in the gene tree associated with that genomic character) it allows us to pose a question about the KVR model: is the KVR model consistent for characters generated by a hypothetical “asymmetric perfect RGC” model? The asymmetric perfect RGC model modifies the perfect model so the ensemble state frequencies at the root differ from the tip frequencies. I recognize that, in addition to the treatment of the root state frequencies, the KVR model differs from parsimony in an important way (specifically, the treatment of branch lengths). However, this conjecture regarding the behavior of the KVR model might point the way toward a falsifiable hypothesis because it lends itself to testing by simulation.

 The question of whether the KVR model is consistent given the asymmetric perfect RGC model is interesting from theoretical standpoint but there is a second (and more important question) that should be answered: is whether the true underlying model of fold content is sufficiently close to the asymmetric perfect RGC model for that model to be useful? The true underlying model of fold evolution includes fold origination (which is almost certainly a very rare event) and horizontal transfer (likely to be much more common). Williams *et al.* (2020) discuss this in their supplementary materials, where they state “…a change from 0 to 1 might indicate de novo origin of an existing fold by convergent evolution (which is likely to be rare), or the gain of an existing fold by [horizontal gene transfer]; if the latter, then the pattern of presences and absences for that fold cannot be reliably used to infer the underlying tree.” I agree with the first part of that sentence (which is a reason why I have invoked the idea of RGCs) but I disagree with the second; even when there is horizontal gene transfer novel fold acquisition might be sufficiently rare for that type of event to be considered an RGC.

 Answering those questions will be challenging and outside the scope of a short note like HM-F1000. In that context, I think it would be good for HM-F1000 to express a little more caution. Statements like “[t]he KVR model is an optimal explanation of the evolution of clade-specific composition of homologous features” (first full paragraph on page 5 of HM-F1000). The point of my arguments above is that it might be reasonable to view the KVR model as an excellent approximating model for protein fold evolution. The first author has written multiple papers dealing with patterns of protein fold evolution over deep evolutionary time and I do not want to disrespect those efforts, but I do not think this is a settled issue at this time.

 II. The best interpretation of the unrooted trees in Williams *et al.* (2020) is unclear.

 The Harish and Kurland (2017) trees are the only intrinsically rooted trees under discussion in Williams *et al.* (2020). Since the position of the root of the 2D-ToL in Williams *et al.* (2020) ultimately represents the imposition of a root on an otherwise unrooted tree. Strictly from a logical standpoint there are four interpretations of the results of Harish and Kurland (2017) and Williams *et al.* (2020).

  

1. Both trees are inaccurate estimates of the ToL.
2. The Harish and Kurland (2017) topology is correct.
3. The unrooted Williams *et al. *(2020) topology is correct and the Harish and Kurland (2017) root is also correct (i.e., the root lies between eukaryotes and akaryotes).
4. The unrooted Williams *et al.* (2020) topology is correct and the root is not between eukaryotes and akaryotes.

 

 Possibility 3 is important, and it would seem to be implicit in Figure 1b of HM-1000. The authors should make this point more explicitly; they use Figure 1b to make another point, using it to stress that “Williams *et al.* have overlooked important aspects of assessing phylogenetic signal in empirical data, and that it may be premature to reject a well-supported phylogeny based on simulated data.” I think it would be valuable to make the point that, at least in principle, the Williams *et al.* (2020) results would be consistent with a tree rooted between eukaryotes and akaryotes that also places the root of akaryotes within the Asgard archaea.

 Obviously, embracing the unrooted Williams *et al.* (2020) topology would require rejecting the akaryote topology of the Harish and Kurland (2017). Specifically, the fact that the Harish and Kurland (2017) tree is consistent with archaeal monophyly (as long as one assumes the root of the tree of life is not within archaea) makes it fundamentally inconsistent with the Williams *et al.* (2020) topology. However, the possibility of archaeal non-monophyly would seem to be consistent with Figures 1 and 2 in Harish (2018) ^13^, both of which show substantial uncertainty at the base of Archaea. Figure 2 in Harish (2018) is based on distances calculated using protein fold data, raising some questions regarding the strength of support for monophyly of Archaea.

 One aspect of the Harish and Kurland (2017) tree that HM-F1000 should acknowledge is the fact plants are not monophyletic. Specifically, the root of the eukaryotic sub-tree of Figure 3 in that paper was placed between rice and all other eukaryotes. Obviously, this is troubling given that the Harish and Kurland (2017) tree includes other angiosperms. In fact, the Harish and Kurland (2017) dataset includes two other grasses; non-monophyly of both angiosperms and grasses is unreasonable. Even placing the eukaryotic root between the green plants and other eukaryotes seems unlikely given the best available information about the eukaryotic tree (reviewed by Burki *et al. *2020) ^14^.

 One might wonder whether the root position for the ToL should be viewed as accurate given the unexpected position of the eukaryotic root. However, it is reasonable to postulate that the rice data were problematic in some way. Alternatively, it could reflect the observation that different data perform differently at different levels in the tree (Chen *et al. *2015) (HM-F1000 already alluded to this). If I had reviewed Harish and Kurland (2017) I would have asked the authors to conduct a second set of analyses after excluding rice to see if that changed the root of the eukaryotic sub-tree. I do not think it would be reasonable to ask HM-F1000 to add a reanalysis of the Harish and Kurland (2017) after excluding rice, but it would be nice for HM-F1000 to acknowledge this issue.

 ---

 Looking back, I realize that I have written this review as an advocate for the position articulated by HM-F1000. Given that tone it would be fair to ask why I not convince that their placement of the root between eukaryotes is accurate? I would answer that question I am not convinced that the tools exist to place of the root of the ToL is accurate exist at this point. As I discussed above, I have made an argument can be made that the KVR model might be able to yield an accurate estimate of the ToL. However, my reasons for that assertion rest on assumptions regarding the nature of the protein fold data. I believe that the Williams *et al.* (2020) analyses do show that the KVR model is imperfect; I simply disagree with their conclusion that this imperfect fit means that we should dismiss the Harish and Kurland (2017) result. Fundamentally, I view the use of the KVR model with protein fold data as similar to the Jukes-Cantor model. Felsenstein (2001) ^15^ shared an anecdote regarding that model, stating that: “Tom Jukes once told me that the reason the Jukes–Cantor model was buried in the midst of a large empirical paper was that this was the only way to get it published. He felt that if he had attempted to publish it on its own, it would have been rejected by editors as idle and oversimplified speculation.” However, without the pioneering work of Jukes and Cantor (1969) ^16^ and Neyman (1971) ^17^ (or Felsenstein 1981) ^18^ it is difficult to envision the development of the more sophisticated models of sequence evolution that developed over the subsequent five decades. Dismissing the use of protein fold data at this point will slow the development of those models. Will further model development support the Eukaryote/Akaryote 2D-ToL? I am uncertain whether it will, but I am interested to find out.

 I would like to add a final discussion regarding model fit. Although there is a long history of model development for protein sequences and the models are now quite sophisticated, there is still much that we do not know about protein evolution. Williams *et al.* (2020) used the LG+C60 model. Presumably this is the C60 profile mixture from Le *et al.* (2008) (although I was unable to find Le *et al. *2008 cited in Williams *et al.* 2020) combined with the LG (Le and Gascuel 2008) ^19^ matrix. However, Pandey and Braun (2020) ^20^ reported that the Le *et al.* (2008) ^21^ profile mixtures can exhibit surprising (and disturbing) behavior for at least one phylogenetic problem. The Le *et al.* (2008) mixture models are very similar to the CAT model (Lartillot and Philippe 2004); it is unclear whether these issues Pandey and Braun (2020) noted for the Le *et al.* (2008) models are general features of CAT-type models (or even more widespread than the particular case studied by Pandey and Braun 2020) but it is important to be careful regarding the use of any models of evolution so deep in the tree.

 It is tempting to look at the sophistication of existing models of protein sequence evolution and conclude that the results obtained using those models trump other sources of information. Although I do not want to be overly dismissive of the Williams *et al.* (2020) analyses, which are state of the art, I do want to emphasize that I believe data type matters and that we should be looking at other sources of information. In my opinion, that is the message that HM-F1000 should convey; that is why I think some statements in HM-F1000, like the statement that the KVR model provides an optimal explanation for protein fold evolution, actually undercut the case. In my opinion, obtaining a strongly corroborated estimate of the deepest branches in the ToL, if it is possible, will require us to examine multiple sources of information and to be very careful regarding the models we use for analyses. That applies to analyses of aligned protein sequences and to analyses of protein fold content. 

 *****

**Section 2: Minor issues and description of necessary revisions:**

  

1. I have written a fairly long review, but I feel the changes to HM-F1000 that are necessary are actually fairly minimal. I think HM-F1000 needs to walk back the claims that the KVR model is an optimal explanation for protein fold distribution and simply point out that it is likely to be a reasonable approximating model. I think HM-F1000 also needs to acknowledge that “both trees could be telling us part of the truth” (i.e., that a tree rooted between eukaryotes and akaryotes with a paraphyletic archaea might be a way to reconcile Harish and Kurland 2017 with Williams *et al.* 2020). HM-F1000 should also acknowledge the unexpected (and incorrect) rooting of the eukaryotic sub-tree. Finally, I hope the minor comments that follow are carefully considered.
2. I was surprised that the work of Poole *et al.* (1998; 1999) ^22^^,^^23^ was not cited. It provides another line of evidence supporting the placement of the root on the eukaryotic branch (i.e., it supports the Eukaryote/Akaryote 2D-ToL).
3. The first full line of the second column of the fourth page of HM-F1000 states “Support from fossils or other sources are not reliable, despite claims to the contrary [Williams *et al.* 2020].” I could not find an explicit statement in Williams *et al.* (2020) that makes this assertion. I agree with the basic point that the fossil record to establish the deep topology for the ToL provides, at most, limited information. However, HM-F1000 should be careful regarding the attribution of statements like this. I hope that I did not miss any such statement in Williams *et al.* (2020); if I have missed it, Harish and Morrison should point to the statement.
4. The legend of Figure 2 states “[t]he majority of proteins are multi-domain proteins formed by duplication and recombination of domain units.” However, Ekman *et al.* (2005) ^24^ reports that fewer than 50% of prokaryotic (akaryotic) proteins are multidomain. I was unable to find an explicit survey of proteins showing that the numbers of multidomain proteins is generally >50% in the literature. This statement should have an associated citation and, if the number is <50% in some lineage be a bit more cautious. Perhaps something like “a large proportion” would be a better statement.
5. The legend of Figure 2 also states that “[a]lthough it is common to suspect that the rooting between akaryotes and eukaryotes could be biased due to a larger domain cohort in eukaryotes [Williams *et al.* 2020], it is not the case.” Since the statement that the large domain cohort of eukaryotes is a source of bias only cites Williams *et al.* (2020) I don’t think it is valid to state that “it is common to suspect” unless there are additional citations. The statement “…it is not the case” cites three papers with Harish as an author, but the evidence that a large domain cohort cannot be a source of bias was not clear to me. The explanation should be expanded a bit and moved to the main text.

Are arguments sufficiently supported by evidence from the published literature or by new data and results?

Yes

Is the conclusion balanced and justified on the basis of the presented arguments?

Partly

Is the rationale for commenting on the previous publication clearly described?

Yes

Are any opinions stated well-argued, clear and cogent?

Yes

Reviewer Expertise:

Evolutionary genomics and computational biology

I confirm that I have read this submission and believe that I have an appropriate level of expertise to confirm that it is of an acceptable scientific standard.

## References

[1] : Phylogenomics provides robust support for a two-domains tree of life. *Nat Ecol Evol*.4(1) : 10.1038/s41559-019-1040-x 138-147 10.1038/s41559-019-1040-x 31819234PMC6942926

[2] : Akaryotes and Eukaryotes are independent descendants of a universal common ancestor. *Biochimie*.2017;138: 10.1016/j.biochi.2017.04.013 168-183 10.1016/j.biochi.2017.04.013 28461155

[3] : A Nonstationary Markov Model Detects Directional Evolution in Hymenopteran Morphology. *Syst Biol*.2015;64(6) : 10.1093/sysbio/syv052 1089-103 10.1093/sysbio/syv052 26272507PMC4604834

[4] : On the impossibility of reconstructing ancestral data and phylogenies. *J Comput Biol*.2003;10(5) : 10.1089/106652703322539015 669-76 10.1089/106652703322539015 14633391

[5] : Testing the Hypothesis of Common Ancestry. *Journal of Theoretical Biology*.2002;218(4) : 10.1006/jtbi.2002.3086 395-408 10.1006/jtbi.2002.3086 12384044

[6] : Rare genomic changes as a tool for phylogenetics. *Trends in Ecology & Evolution*.2000;15(11) : 10.1016/S0169-5347(00)01967-4 454-459 10.1016/S0169-5347(00)01967-4 11050348

[7] : Rare Genomic Changes.2017; 10.1007/978-3-319-54064-1_10 195-211 10.1007/978-3-319-54064-1_10

[8] : Two further links between MP and ML under the poisson model. *Applied Mathematics Letters*.2004;17(7) : 10.1016/j.aml.2004.06.006 785-790 10.1016/j.aml.2004.06.006

[9] : Maximum parsimony and the phylogenetic information in multistate characters.2006; 10.1093/acprof:oso/9780199297306.003.0009 163-178 10.1093/acprof:oso/9780199297306.003.0009

[10] : Parsimony, likelihood, and simplicity. *Cladistics*.2003;19(2) : 10.1111/j.1096-0031.2003.tb00297.x 91-103 10.1111/j.1096-0031.2003.tb00297.x

[11] : A Bayesian perspective on a non-parsimonious parsimony model. *Syst Biol*.2008;57(3) : 10.1080/10635150802166046 406-19 10.1080/10635150802166046 18570035

[12] : Phylogenetic analysis using parsimony and likelihood methods. *J Mol Evol*.1996;42(2) : 10.1007/bf02198856 294-307 10.1007/bf02198856 8919881

[13] : What is an archaeon and are the Archaea really unique?. *PeerJ*.2018;6: 10.7717/peerj.5770 e5770 10.7717/peerj.5770 30357005PMC6196074

[14] : The New Tree of Eukaryotes. *Trends Ecol Evol*.35(1) : 10.1016/j.tree.2019.08.008 43-55 10.1016/j.tree.2019.08.008 31606140

[15] : Taking variation of evolutionary rates between sites into account in inferring phylogenies. *J Mol Evol*.53(4-5) : 10.1007/s002390010234 447-55 10.1007/s002390010234 11675604

[16] : Evolution of Protein Molecules.1969; 10.1016/B978-1-4832-3211-9.50009-7 21-132 10.1016/B978-1-4832-3211-9.50009-7

[17] : MOLECULAR STUDIES OF EVOLUTION: A SOURCE OF NOVEL STATISTICAL PROBLEMS**This investigation was supported in part by research grant GM 10525-08 from the National Institutes of Health, Public Health Service. 1971; 10.1016/B978-0-12-307550-5.50005-8 1-27 10.1016/B978-0-12-307550-5.50005-8

[18] : Evolutionary trees from DNA sequences: a maximum likelihood approach. *J Mol Evol*.1981;17(6) : 10.1007/bf01734359 368-76 10.1007/bf01734359 7288891

[19] : An improved general amino acid replacement matrix. *Mol Biol Evol*.2008;25(7) : 10.1093/molbev/msn067 1307-20 10.1093/molbev/msn067 18367465

[20] : Phylogenetic Analyses of Sites in Different Protein Structural Environments Result in Distinct Placements of the Metazoan Root. *Biology*.2020;9(4)(64) : 10.3390/biology9040064 10.3390/biology9040064 PMC723575232231097

[21] : Empirical profile mixture models for phylogenetic reconstruction. *Bioinformatics*.2008;24(20) : 10.1093/bioinformatics/btn445 2317-23 10.1093/bioinformatics/btn445 18718941

[22] : The path from the RNA world. *J Mol Evol*.1998;46(1) : 10.1007/pl00006275 1-17 10.1007/pl00006275 9419221

[23] : Early evolution: prokaryotes, the new kids on the block. *BioEssays*.1999;21(10) : 10.1002/(SICI)1521-1878(199910)21:10<880::AID-BIES11>3.0.CO;2-P 880-889 10.1002/(SICI)1521-1878(199910)21:10<880::AID-BIES11>3.0.CO;2-P 10497339

[24] : Multi-domain proteins in the three kingdoms of life: orphan domains and other unassigned regions. *J Mol Biol*.2005;348(1) : 10.1016/j.jmb.2005.02.007 231-43 10.1016/j.jmb.2005.02.007 15808866

# Response

**Published:** 2020-06-15

**Authors:** Ajith Harish

**Affiliations:** Unaffiliated, Sweden

**Response to reviewer**

We thank the reviewer for their detailed review and thoughtful comments. We agree with the issues raised by the reviewer. We revised the text accordingly.

**Suggestion:** I have written a fairly long review, but I feel the changes to HM-F1000 that are necessary are actually fairly minimal. I think HM-F1000 needs to walk back the claims that the KVR model is an optimal explanation for protein fold distribution and simply point out that it is likely to be a reasonable approximating model. I think HM-F1000 also needs to acknowledge that “both trees could be telling us part of the truth” (i.e., that a tree rooted between eukaryotes and akaryotes with a paraphyletic archaea might be a way to reconcile Harish and Kurland 2017 with Williams et al. 2020).

**Response:** We have included the following in the second to last paragraph of the manuscript,

“Even if the archaeal radiation remains poorly resolved with more data, the better supported rooting between eukaryotes and akaryotes is consistent with a Eukaryote-Akaryote 2D-ToL (Figure 1b). That is, diversification of eukaryotes and akaryotes from the UCA is a better supported hypothesis rather than a prokaryote-to-eukaryote transition being assumed to interpret poorly resolved trees.”

    

**Suggestion: **I was surprised that the work of Poole et al. (1998; 1999)22,23 was not cited. It provides another line of evidence supporting the placement of the root on the eukaryotic branch (i.e., it supports the Eukaryote/Akaryote 2D-ToL).

**Response: **We have now cited the suggested article in our discussion about the relative age of eukaryotes and akaryotes in the last paragraph of section 1.

    

**Suggestion: **The first full line of the second column of the fourth page of HM-F1000 states “Support from fossils or other sources are not reliable, despite claims to the contrary [Williams et al. 2020].” I could not find an explicit statement in Williams et al. (2020) that makes this assertion. I agree with the basic point that the fossil record to establish the deep topology for the ToL provides, at most, limited information. However, HM-F1000 should be careful regarding the attribution of statements like this. I hope that I did not miss any such statement in Williams et al. (2020); if I have missed it, Harish and Morrison should point to the statement.

**Response: **The reference was to the Williams et al (2020) statement “ *At present, the best-supported root is on the branch separating bacteria and archaea ^67,68,80,81^ or among the bacteria ^70,72,^ and the hypothesis that eukaryotes are younger than prokaryotes is supported by a range of phylogenetic, cell biological ^2,3^ and palaeontological ^61,82–84^ evidence.*”

We have revised our statement for clarity, also related to the discussion of the relative age of eukaryotes and akaryotes as: “Thus, the location of the archaeal-CA or UCA remains ambiguous at best (Figure 1b), regardless of the gene-aggregation and tree-reconciliation method used for estimating a consensus unrooted tree… Despite claims to the contrary, that the best-supported root is on the branch separating bacteria and archaea or that eukaryotes are younger than akaryotes7, support from fossils is not reliable either, since assigning fossils to extinct archaea/bacteria or UCA is even more ambiguous.”

**Suggestion: **The legend of Figure 2 states “[t]he majority of proteins are multi-domain proteins formed by duplication and recombination of domain units.” However, Ekman et al. (2005)24 reports that fewer than 50% of prokaryotic (akaryotic) proteins are multidomain. I was unable to find an explicit survey of proteins showing that the numbers of multidomain proteins is generally >50% in the literature. This statement should have an associated citation and, if the number is <50% in some lineage be a bit more cautious. Perhaps something like “a large proportion” would be a better statement.

**Response: **The statement now reads “a large proportion” as suggested.

    

**Suggestion: **The legend of Figure 2 also states that “[a]lthough it is common to suspect that the rooting between akaryotes and eukaryotes could be biased due to a larger domain cohort in eukaryotes [Williams et al. 2020], it is not the case.” Since the statement that the large domain cohort of eukaryotes is a source of bias only cites Williams et al. (2020) I don’t think it is valid to state that “it is common to suspect” unless there are additional citations.

**Response: **We have revised the statement as “ Despite the suspicion that the rooting between akaryotes and eukaryotes could be biased due to a larger domain cohort in eukaryotes …. ”

**Suggestion: **The statement “…it is not the case” cites three papers with Harish as an author, but the evidence that a large domain cohort cannot be a source of bias was not clear to me. The explanation should be expanded a bit and moved to the main text.

**Response: **We included a short discussion in the third to last paragraph of section 1 as: “. In contrast, the separation of eukaryotes and akaryotes (and of archaea and bacteria) is unperturbed even after extreme perturbation of the domain composition in eukaryotes (e.g. by excluding up to two-thirds of the domain cohort, Figure 2b). The clades within eukaryotes and akaryotes are unperturbed as well ^11^".
